# Plant-Derived Natural Antioxidants in Meat and Meat Products

**DOI:** 10.3390/antiox9121215

**Published:** 2020-12-02

**Authors:** Georgios Manessis, Aphrodite I. Kalogianni, Thomai Lazou, Marios Moschovas, Ioannis Bossis, Athanasios I. Gelasakis

**Affiliations:** 1 Laboratory of Anatomy and Physiology of Farm Animals, Department of Animal Science, Agricultural University of Athens (AUA), Iera Odos 75 Str., 11855 Athens, Greece; gmanesis@aua.gr (G.M.); afrokalo@aua.gr (A.I.K.); moschovas@aua.gr (M.M.); 2 Laboratory of Hygiene of Foods of Animal Origin-Veterinary Public Health, School of Veterinary Medicine, Faculty of Health Sciences, Aristotle University of Thessaloniki, 54124 Thessaloniki, Greece; tlazou@vet.auth.gr; 3 Laboratory of Animal Husbandry, Department of Animal Production, Faculty of Agriculture, Forestry and Natural Environment, School of Agriculture, Aristotle University of Thessaloniki, 54124 Thessaloniki, Greece; bossisi@agro.auth.gr

**Keywords:** natural antioxidants, antioxidant activity, spices and herbs, fruit-derived antioxidants, lipid oxidation, protein oxidation, meat, meat-based products

## Abstract

The global meat industry is constantly evolving due to changes in consumer preferences, concerns and lifestyles, as well as monetary, geographical, political, cultural and religious factors. Part of this evolution is the introduction of synthetic antioxidants to increase meat and meat products’ shelf-life, and reduce meat spoilage due to lipid and protein oxidation. The public perception that natural compounds are safer and healthier per se has motivated the meat industry to replace synthetic antioxidants with plant-derived ones in meat systems. Despite several promising results from in vitro and in situ studies, the effectiveness of plant-derived antioxidants against lipid and protein oxidation has not been fully documented. Moreover, the utility, usability, marketability and potential health benefits of natural antioxidants are not yet fully proven. The present review aims to (i) describe the major chemical groups of plant-derived antioxidants and their courses of action; (ii) present the application of spices, herbs and fruits as antioxidants in meat systems; and (iii) discuss the legislative framework, future trends, challenges and limitations that are expected to shape their acceptance and mass exploitation by the meat industry.

## 1. Introduction

Meat and related products are excellent sources of proteins and amino acids, fats, minerals (e.g., zinc, iron and phosphorus), vitamins, and other valuable or essential nutrients; therefore, they constitute an integral part of human nutrition [1]. Despite emerging dietary trends in Western societies that promote the reduction and/or replacement of meat in human diets [2], global meat consumption is constantly increasing; over the last 20 years, it has increased by 58%, reaching 360 million tons annually, with 54% of this increase being attributed to population growth, and the rest being ascribed to increased consumption per capita as shaped by changes in consumers’ dietary attributes and incomes [3]. In financial terms, the value of the meat sector worldwide was USD 945.7 billion in 2018 and is expected to reach USD 1142.9 billion in 2023 [4].

Worldwide, the meat industry has been evolving, driven by consumer preferences, concerns and lifestyles, as well as by monetary, geographical, political, cultural and religious factors. This evolution has been linked with increased demands for extended meat preservation, which traditionally forms the pillar of international meat trade and is directly associated with meat safety and quality assurance. Techniques such as salting, drying, smoking, fermentation and canning have traditionally been used to extend meat shelf life [5]. Today, meat preservation techniques have been expanded with the universal use of (i) cold chain logistics, (ii) novel thermal treatments (superchilling, ultrarapid freezing, immersion vacuum cooling, pressure-shift freezing, dielectric heating and ohmic heating), (iii) advances in packaging (modified atmospheres, vacuums, novel materials etc.), and (iv) the addition of preservatives, which have been utilized to enhance the safety and quality of meat and meat products [6,7,8]. Despite the exploitation of the aforementioned preservation strategies, significant meat losses are still observed across the food supply chain (from farm to fork), especially in developing regions. In industrialized regions, losses mainly occur at the market and household level, rather than at the farm and processing plant level [9].

Lipid and protein oxidation in meat products is the second most important cause of meat spoilage (following microbial spoilage) [10]. It is associated with intrinsic and extrinsic quality degradation, reduced nutritional value and a general deterioration in meat palatability and other organoleptic traits [11,12,13]. Meat oxidation is related to its increased content of proteins, fats, carbohydrates and water [5] and results in the formation of chemical compounds with cytotoxic, mutagenic and oxidative effects on body tissues, thereby promoting cancer, atherosclerosis, inflammation and aging processes [12]. For example, lipid peroxides are associated with oxidative damage to colon cells and inflammation and cancer in the digestive tract [14], cholesterol oxidation products (COPs) disrupt the junctions between arterial endothelial cells and are related to atherosclerosis and coronary diseases [15], and aldehydes and oxysterols exhibit cytotoxic and mutagenic action in different types of cells, contributing to the manifestation of chronic diseases and cancer [16].

To avert the effects of oxidation in meat and meat products, chemical antioxidants are extensively used. The chemical compounds utilized for their antioxidant properties include (i) phenol derivatives such as butylated hydroxyanisole (BHA), butylated hydroxytoluene (BHT), tertiary butylhydroquinone (TBHQ), propyl gallate (PG) and synthetic tocopherol (vitamin E), with both groups exhibiting free-radical scavenging activity [17]; (ii) phosphates (e.g., tripolyphosphate, hexametaphosphate and pyrophosphate etc.), which chelate divalent cations and bind iron, retarding rancidity and oxidation; and (iii) acids (organic, HCl), which obstruct calpain, cathepsins and aminopeptidase enzymes, which are responsible for the enzymatic deterioration of meat and meat products [5]. The aforementioned chemical antioxidants can be jointly used with chemical preservatives (e.g., nitrites, sulfites, benzoic acid and sorbic acid).

Currently, the awareness of the possible health implications of the extended use of synthetic antioxidants (allergic reactions, disorders in pregnant women and children, potential carcinogenic action etc.) [18] has promoted consumer-driven demands supporting the use of natural antioxidant compounds. Although the allowed content of synthetic antioxidants in the lipidic fraction is low (0.02% w/w) and they are therefore safe for consumers when used in accordance with the relevant regulations [19], the public opinion that natural compounds are safer and health-beneficial per se has motivated the meat industry to exploit plant-derived additives in meat systems with the objective of replacing synthetic antioxidants [20].

The objectives of the present literature review were (i) to summarize the knowledge about plant-derived antioxidants and their applications in meat systems, emphasizing the mechanisms and the spectrum of their antioxidant activity, and (ii) to discuss the legislative framework, trends, limitations and challenges for their mass utilization by the meat industry.

## 2. Lipid and Protein Oxidation of Meat

Reactive Oxygen Species (ROS) and Reactive Nitrogen Species (RNS) are free radicals normally found in living tissues. Their contents are modulated by oxidation–reduction reactions that participate in the regulation of cellular homeostasis [21]. The absence of post-mortem homeostatic mechanisms in muscles allows ROS and RNS to accumulate in meat and to interact with proteins, fatty acids and nucleic acids, resulting in the generation of oxidation by-products, which have detrimental effects on meat quality, safety and preservation potential [10].

**Figure 1:**
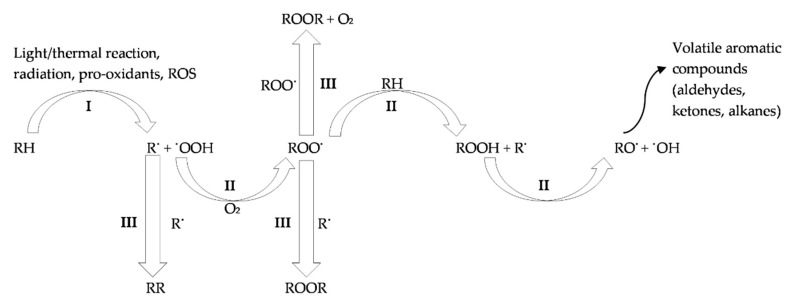
Mechanism of lipid oxidation. I: initiation, II: propagation, III: completion, RH: unsaturated fatty acid, ROS: reactive oxygen species, R^●^: alkyl radical, RR: non-radical product, ROO^●^: peroxide radical, ROOR: organic peroxide, ROOH: hydroperoxides, ^●^OOH: hydroperoxyl radical, RO^●^: alkoxy radical, ^●^OH: hydroxyl radical, O_2_: oxygen.

Lipid oxidation is the result of free-radical chain reactions induced by metallic ions (heme iron), ROS and RNS [22]. Chain reactions of lipid oxidation occur in three phases: (i) initiation, (ii) propagation and (iii) completion (Figure 1). During the first two phases, factors such as the generation of reactive compounds, exposure to oxygen and ultraviolet light, the disruption of cell integrity, the presence of pro-oxidant metal ions and exposure to gamma irradiation favor the production of free radicals [23]. The produced free radicals are rapidly transformed into non-radical compounds such as conjugated dienes (carbon chains possessing two double bonds separated by a single bond) and hydroperoxides (ROOH), which are the primary products of lipid oxidation [12,24]. The decomposition of these compounds, at the last phase of oxidation, generates secondary products such as aldehydes, ketones, alcohols and carbonyl compounds [12,24]. Aldehydes (e.g., alkenals, alkadienals and hydroxyalkenals) are key secondary products due to their rapid reactions with proteins, which results in undesirable modifications of meat’s organoleptic and nutritional traits [24]. Moreover, α,β-unsaturated aldehydes (crotonaldehyde, malondialdehyde, acrolein, 4-hydroxy-transnonenal and 4-hydroxy-trans-hexanal), alcohols and oxidized cholesterol constitute toxic products of oxidation and have been found to be associated with health problems such as inflammatory diseases, cancer and atherosclerosis [25]. The main methods used to assess the oxidation of fats are the peroxide value, thiobarbituric acid reactive substances (TBARS) analysis and chromatography, while novel methodologies such as chemiluminescence, fluorescence emission, Raman spectroscopy, infrared spectroscopy and magnetic resonance provide promising results [26,27]. 

**Figure 2:**
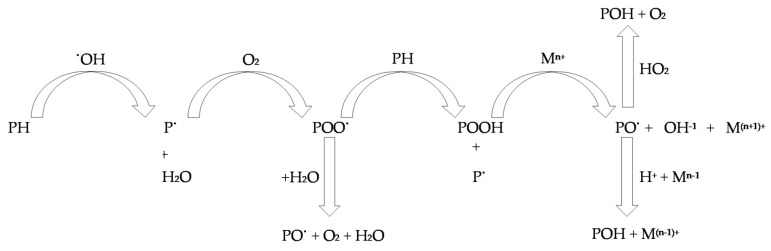
Mechanism of protein oxidation. PH: protein, ^●^OH^.^: hydroxyl radical, P^●^: protein carbon-centered radical, H_2_O: water, O_2_: oxygen, POO^●^: analkylproxyl radical, PO^●^: alkoxyl radical, POOH: alkylperoxide, M^n+^: reduced forms of transition metals (Fe^2+^ or Cu^1+^), OH^−1^: hydroxide, POH: hydroxyl derivative, HO_2_: hydroperoxyl, H^+^: hydron.

Protein oxidation (Figure 2) is initiated by myoglobin, metallic catalysts or oxidizing lipids, which react with side chains of amino acids, eventually leading to the creation of protein radicals and carbonyl derivatives and causing protein carbonylation [24]. The major determinants of the oxidative process outcome are (i) the fatty acid profile and content, (ii) the type of antioxidant compounds (vitamins, enzymes or peptides) and (iii) the contents of pro-oxidant compounds such as heme-protein pigments, metals and enzymes [12,28]. Several assays are used to assess the oxidation of proteins in meat; the major oxidation indicators utilized by these assays are protein carbonyl (e.g., α-amino adipic semialdehyde (AAS) and γ-glutamic semialdehyde (GGS)) and free thiol contents, bityrosine production, protein radical intensity, amounts of proteins and myoglobin, non-heme-iron content and lipoxygenase activity [24,29,30].

## 3. Antioxidants Used in Meat Systems

To mitigate the effects of free radicals in meat systems, compounds with antioxidant activity are utilized. These compounds are characterized as “antioxidants”, a term that is currently used to describe any substances that delay or prevent the oxidation of biomolecules in food, even when added at low concentrations [31].

The antioxidants exploited by the meat industry are either natural or synthetic. Natural antioxidants can be further classified based on their origin (plants, animals or bacteria) and their chemical structure (e.g., phenols, tocopherols or vitamin C). Plant-derived natural antioxidants originate from fruits, teas and herbs, seeds, spices, vegetables, cereals and trees [32]. Various plant organs such as leaves, flowers, fruits, stems or roots accumulate antioxidants in high concentrations, which vary according to the plant species and the antioxidant substance itself. The aforementioned plant parts are used in food products either directly (e.g., fruit puree or juice) or after the extraction and purification of the antioxidant substances they contain (e.g., rosemary extract) [20,33].

**Figure 3:**
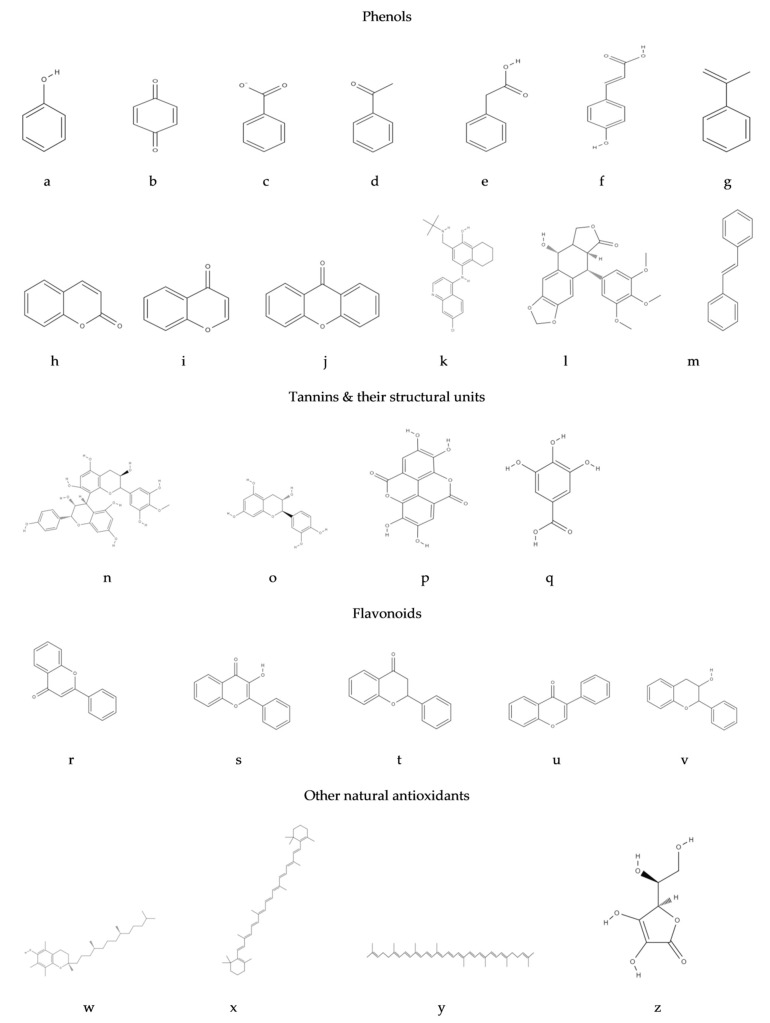
Basic chemical structures of selected phenolic antioxidants. Phenols: (**a**) phenol, (**b**) benzoquinone, (**c**) phenolic acid, (**d**) acetophenone, (**e**) phenylacetic acid, (**f**) hydroxycinnamic acid, (**g**) 2-phenylpropene, (**h**) coumarin, (**i**) chromone, (**j**) xanthone, (**k**) naphthoquinone, (**l**) lignans, (**m**) stilbene. Tannins: (**n**) proanthocyanidins, (**o**) catechin, (**p**) ellagic acid, (**q**) gallic acid. Flavonoids: (**r**) flavone, (**s**) flavanol, (**t**) flavanone, (**u**) isoflavone, (**v**) flavan-3-ol. Other natural antioxidants: (**w**) α-tocopherol, (**x**) β-carotene, (**y**) lycopene, (**z**) vitamin C.

The chemical structure of antioxidants is related to their properties and their main course of action, while the major groups of antioxidant substances based on their chemical structures are phenols, tannins, flavonoids and isoflavonoids, anthocyanins, lignans, stilbenes, tocopherols, carotenoids and vitamin C and are presented in Figure 3. 

### 3.1. Phenols

Phenols are secondary products of plant metabolism derived from the aromatic amino acid phenylalanine, through the shikimic acid (phenylpropanoids) and the acetic acid (simple phenols) pathways; phenols contribute to the color, flavor and astringency of plants [34]. Phenolic compounds can be classified based on their (i) plant species origin, (ii) chemical structure (the number and arrangement of hydroxyl moieties, double bonds in the carbon rings, and type and degree of alkylation and/or glycosylation) [35] and (iii) solubility in water, which affects their nutritional, metabolic and physiological action; their common feature is a hydroxy-substituted benzene ring within their structure [36]. Phenolic compounds act as hydrogen donors [36], thus quenching free radicals, or transfer single electrons to reduce chemical compounds with oxidative action. Apart from their antioxidant capacity, phenolic compounds have an important functional and biological role in plant physiology, acting as structural polymers, UV radiation screeners, pollination and soil bacterium attractants, non-specific defense mechanisms, phytoalexins, and potentially signal compounds for systemic acquired resistance [36]. Flavan monomers, dimers and polymers, and cinnamic acid derivatives form the major fraction of natural phenolic compounds [34], which, in general, are categorized into (i) phenols and benzoquinones; (ii) phenolic acids; (iii) acetophenones and phenylacetic acids; (iv) hydroxycinnamic acids, phenylpropenes, coumarins-isocoumarins and chromones; (v) naphthoquinones; (vi) xanthones; (vii) stilbenes and anthraquinones; (viii) flavonoids; (ix) lignans and neolignans; and (x) lignins (Table 1).

#### 3.1.1. Tannins

Tannins are water-soluble, astringent, polyphenolic substances, produced by the polymerization of phenylpropanoid compounds [34]. Traditionally, tannins were used for protein precipitation due to their ability to interact with at least two protein molecules and form insoluble, cross-linked tannin–protein complexes [34]. Tannins are classified into (i) condensed tannins (proanthocyanidins), which are polymers of catechin, epicatechin, prodelphinidins, profisetinidins and prorobinetidins, and (ii) hydrolysable tannins, which can be hydrolyzed by weak acids or bases, with the latter being mixtures of carbohydrates with gallic and ellagic acid (gallotannins and ellagitannins) [55]. Proanthocyanidins donate hydrogen and electrons (primary antioxidant action), chelate ferrum and inhibit the activity of cyclooxygenase (secondary antioxidant action) [56]. The consumption of small quantities of tannins has a positive effect on lipid metabolism and the regulation of immune responses, reduces blood pressure and presents anticarcinogenic and antimutagenic activity [57]. Nevertheless, foods rich in tannins have lower nutritional value due to the ability of tannins to flocculate proteins, decreasing their digestibility [57].

#### 3.1.2. Flavonoids

Flavonoids and isoflavonoids can be found in various plants (Table 1) and derive from the aromatic amino acids phenylalanine and tyrosine, and from malonate. The basic structure of flavonoids is the flavan nucleus, which consists of three rings of carbon atoms (C6-C3-C6). The level of oxidation and the pattern of carbon atom ring substitution are used to discriminate the classes of flavonoids (flavones, flavanones, isoflavones, flavonols, flavanonols, flavan-3-ols, anthocyanidins, biflavones, chalcones, aurones and coumarins) [58]. Flavonoid antioxidant action includes (i) the suppression of the formation of ROS by inhibiting enzymatic reactions and chelating elements involved in free-radical production, and (ii) the scavenging of ROS [59]. Flavonoids have shown significant antioxidant capacity during in vitro experiments and are considered to be associated with a decreased risk of developing cardiovascular diseases, hypertension, Alzheimer’s disease and certain types of cancer [58,60]. However, the enteric absorption of flavonoids in humans is limited; therefore, these benefits possibly remain underexploited [60].

#### 3.1.3. Lignans

Lignans are diphenol compounds belonging to phytoestrogens. They derive from the amino acid phenylalanine, following the dimerization of substituted cinnamic alcohols [56,61]. Lignans act as hydrogen donors and complex divalent transition metal cations, which explains their antioxidant activity [62]. The main sources of lignans are linseed, pumpkin and sesame seeds, broccoli, soybeans and some types of berries [62]. Secoisolariciresinol diglucoside, the main lignan of linseeds, is converted to enterodiol and enterolactone by the colon microflora, exhibiting preventive action against atherosclerosis [63] and colon, breast, endometrial and prostate cancer [62,64]. Sesame lignans enhance the antioxidant activity of vitamin E in lipid peroxidation systems and increase its radical scavenging activity (sesamin) [56].

#### 3.1.4. Stilbenes

Stilbenes are characterized by a 1,2-diphenylethylene structure. They are classified into monomeric and oligomeric stilbenes [65], and their main sources in human diets are grapes and wine, peanuts and some types of berries [66]. Stilbenes have a beneficial effect on chronic diseases such as cancer [67], cardiovascular and neurodegenerative pathologies by modulating redox status, cell proliferation, mitochondrial activity and the production of inflammation markers [66]. Resveratrol in red wine is the most studied stilbene due to its beneficial effect on heart health. Although resveratrol bioavailability is reduced through metabolic processes and under the effect of the intestinal microflora, its metabolites are still associated with specific health benefits [66].

### 3.2. Vitamin E

Vitamin E group compounds include tocopherols and tocotrienols. They are phenolic substances synthesized by plants (e.g., cabbage, oregano and paprika, etc.) and are significant components in human and animal diets [68]. Vitamin E group compounds have a chroman head, consisting of a phenolic and a heterocyclic ring, conjugated with a phytyl chain [68]. Tocopherols occur in four homologues, α, β, γ and δ, discriminated by the number of methyl substituents of the saturated phytyl chain and the substitution patterns of the phenolic ring [69]. Tocotrienols differentiate from tocopherols by the three trans- double bonds in their hydrocarbon tails [70]. The antioxidant activity of tocopherols relies on their ability to donate hydrogen to lipid free radicals, and their antioxidant capacity has been demonstrated both in vitro and in vivo. In both types of studies, the highest antioxidant action was observed for α-tocopherol, followed by the homologues β, γ and δ [68]. The highest antioxidant capacity of α-tocopherol, in vivo, is exhibited due to its preferential retention and distribution in animal species [71]. Vitamin E compounds protect membrane lipids by scavenging peroxy-radicals and quenching or interacting with singlet oxygen ^1^O_2_ or ROS [71]. Simultaneously, the oxidation products of vitamin E compounds are considered as pro-oxidants that propagate peroxidation, if not reduced by vitamin C [72]. Tocopherols have beneficial effects on protein kinase C and protein phosphatase 2 activity, as well as on gene expression and cell proliferation, while tocotrienols exhibit neuroprotective, anti-cancer and cholesterol-lowering effects [70,72]. These effects are partially if not exclusively attributed to the antioxidant activity of vitamin E compounds [73].

### 3.3. Carotenoids

Carotenoids are lipophilic, pigmented compounds naturally synthesized by microorganisms and plants (e.g., acerola, cabbage, carrots and paprika) but not animals. Carotenoids share a polyisoprenoid structure, consisting of a carbon chain of conjugated carbon bonds, and possess a near-bilateral symmetry around the central double bond [74]. Cyclic end-groups are attached at the central chain and can be substituted with functional groups that contain oxygen [75]. Based on the type of substitution, carotenoids are further classified into carotenes (e.g., β-carotene and lycopene, which contain only carbon and hydrogen atoms) and oxycarotenoids (xanthophylls), which contain at least one oxygen atom [75]. The most studied carotenoids are lycopene (with eleven conjugated double bonds and two acyclic end-groups) and β-carotene (with eleven conjugated double bonds and two cyclohexene type groups) [76]. The antioxidant capacity of carotenoids is dependent on the number of conjugated double bonds and is manifested by their ability to scavenge singlet oxygen ^1^O_2_ and peroxyl-radicals by physical quenching [75]. Moreover, carotenoids have provitamin A activity and are associated with the regulation of lipoxygenases and connexin 43 gene expression [76]. Carotenoids exhibit beneficial action against cataracts and macular degeneration [77] and have been found to reduce the risk of some cancers (e.g., lung cancer) in the general population. The administration of increased doses of carotenoids to high-risk population groups, such as smokers and asbestos workers, increases the risk of developing lung cancer [78].

### 3.4. Vitamin C

Vitamin C or L-ascorbic acid is a six-carbon lactone. It is synthesized from glucose and is considered as a vitamin for only a few vertebrate species (humans, primates and guinea pigs) due to the deficiency of l-gulonolactone oxidase caused by mutations in the enzyme’s gene [79,80]. Vitamin C functions as an electron donor, thus scavenging free radicals and preventing the oxidation of other molecules; it is capable of donating two electrons from a double bond located between C2 and C3 of the six-carbon chain [79]. Vitamin C is well-known for the prevention and treatment of scurvy. Moreover, it has a positive effect on the innate and adaptive immune systems by supporting the cellular functions of neutrophils and epithelial barrier cells and the differentiation and production of B- and T-cells [81]. Vitamin C has an important role in iron absorption, the regulation of hypertension and the prevention of vascular disease and gastric cancer [79]. Despite its significant antioxidant capacity observed by in vitro studies, epidemiological data and in vivo studies show that, in healthy individuals, it is not likely to have extra health benefits when the daily intake exceeds the recommended [31,79,82]. In the latter case, excessive vitamin C is excreted through urine, due to its hydrophilic properties and the low vitamin-storage capabilities of cells and tissues.

## 4. Course of Action of Antioxidants

Antioxidants exhibit various courses of action and biokinetics, which are determined by their chemical structures and interaction modes, as well as by the physiological factors of animals and physicochemical traits of meat. The courses of action of antioxidants include (i) the inhibition of chain-reaction initiation by scavenging oxidation-initiating radicals, (ii) the breaking of chain reactions that abstract hydrogen for prolonged times, (iii) the decreasing of localized oxygen concentrations, (iv) the decomposition of peroxides and the prevention of their conversion to initiation radicals, and (v) the chelation of chain-reaction initiating catalysts, such as metal ions [19,20,83]. 

Antioxidants are classified into five categories based on their courses of action. (i) Primary antioxidants terminate the free-radical chain reactions by donating electrons or hydrogen. Primary antioxidants include phenolic compounds, tocopherols and synthetic antioxidants such as alkyl gallates, BHA, BHQ and TBHQ. (ii) Oxygen scavengers react with oxygen and reduce it in closed systems; they include vitamin C, ascorbyl palmitate, erythorbic acid and its sodium salt. (iii) Secondary antioxidants decompose lipid hydroperoxides into stable end-products such as dilauryl thiopropionate and thiodipropionic acid. (iv) Enzymatic antioxidants remove oxygen (e.g., glucose oxidase) or ROS (e.g., superoxide dismutase, catalase etc.). (v) Chelating agents capture metallic ions such as copper and iron that act as catalysts of lipid oxidation. Some common chelating agents are citric acid, ethylenediaminetetraacetic acid (EDTA) and amino acids, which may also exhibit synergistic action with phenolic antioxidants [19,84].

The complexity of metabolism and the oxidative pathways in animal tissues, the scarcity of wide-scale studies on the effects of antioxidants on human health, the contradictory literature (e.g., the case of β-carotene [85]) and the variety of antioxidants contained in food systems do not allow the efficient extrapolation of the results from in vitro studies to fit into in situ applications on an evidentiary basis.

## 5. Assays Used to Assess Antioxidant Capacity of Meat and Its Products

Several assays are used to measure the antioxidant capacity of meat and its products. They include (i) assays for the determination of TBARS, (ii) assays to measure hydrophilic antioxidants (Trolox equivalent antioxidant capacity (TEAC), 2,2-diphenyl-1-picrylhydrazyl (DPPH), *β*-carotene bleaching (BCB), superoxide radical scavenging activity (SRSA), total radical-trapping antioxidant parameter (TRAP)/oxygen-radical absorbance capacity (ORAC), photochemiluminescence (PCL), chemiluminescence (CL), ferric reducing antioxidant power (FRAP), the ferric thiocyanate method (FTC), hydroxyl radical scavenging capacity (HOSC), electron spin resonance (ESR)/electron paramagnetic resonance (EPR), electrochemical methods, and the ferrous oxidation xylenol orange (FOX) assay), (iii) assays to measure lipophilic antioxidants (TEAC, lipophilic oxygen radical absorbance capacity (ORAC), and ORAC with randomly methylated *β*-cyclodextrin), (iv) the Rancimat method and (v) the measurement of the activity of antioxidant enzymes (superoxide dismutase (SOD), catalase (CAT) and glutathione peroxidase (GSHPx)) [86].

The plethora of the available methods obstructs benchmarking capabilities for the assessment of the antioxidant capacity of foods. The standardization of analytical protocols would facilitate the proper use of assays to provide robust data for comparisons between antioxidants and their efficient application in meat systems [87]. An ideal assay should be simple, detect both hydrophilic and lipophilic antioxidants, utilize a biologically relevant radical source and a well-defined mechanism, provide reproducible results, be high-throughput and exploit commercially available instruments [87]. The main limitation of the aforementioned methods, when used to test complex samples (that usually contain more than one antioxidant), is that they are not substance-specific, and therefore, an isolation/extraction step is required to assess the activity of individual antioxidant substances. The most commonly used methods for the assessment of antioxidants in meat systems are TBARS, TEAC, ORAC, DPPH, FRAP and the Folin–Ciocalteu assay.

Among the aforementioned methods, TBARS is the method most widely used for the measurement of antioxidant capacity in meat samples based on the estimation of malondialdehyde (MDA) content [88]. MDA is one of the major end-products of lipid oxidation and is associated with the development of undesired odors in meat [88]. MDA reacts with thiobarbituric acid (TBA), resulting in the formation of a pink adduct; this phenomenon, combined with visible spectrometry, is utilized by the TBARS assay for the quantification of MDA and the assessment of the antioxidant capacity of a compound (the addition of a compound with antioxidant activity adversely affects the formation of the pink pigment). Although MDA is not the exclusive product of lipid oxidation capable of reacting with TBA, the use of the assay is widely accepted by the meat industry. The main drawbacks of the method are the low reproducibility and the time required for its completion [88,89].

TEAC is a decolorization assay, applicable to both lipophilic and hydrophilic antioxidants, based on the scavenging of the 2,2′-azinobis-(3-ethylbenzothiazoline-6-sulfonate) radical (ABTS˙) by hydrogen-donating antioxidants [90]. The method has two stages: (i) the generation of the ABTS˙ radical chromophore through the reaction with potassium persulfate, and (ii) the addition of antioxidants that reduce ABTS˙ radicals and alter the absorbance. This reduction is related to the antioxidant activity, the concentration of the antioxidant and the duration of the reaction. The extent of the decolorization of the ABTS˙ radical is determined as a function of concentration and time, and compared with the reactivity of Trolox under the same conditions [90]. TEAC is user-friendly, fast, inexpensive and not affected by pH, but requires an extra step for the creation of the radical; the radical deteriorates during extensive periods of time, and the method is not standardized; therefore, data comparison is problematic [91].

ORAC utilizes an indicator protein, 2,2′-azobis(2-amidinopropane) dihydrochloride (AAPH) as a peroxyl radical generator, and Trolox as a control. The decrease in the fluorescence emission of an excited protein is a result of the oxidative damage caused by the source of peroxyl radicals, which affects the protein structure. The method correlates the ability of the antioxidants to protect the protein from oxidative damage with that of the synthetic antioxidant Trolox [92]. The protein initially used was β-phycoerythrin (β-PE), but disadvantages, such as the inconsistency between batches, photosensitivity and interaction with phenolic compounds, has led to the substitution of β-PE with 3′,6′-dihydroxyspiro[isobenzofuran-1[3H],9′[9H]-xanthen]-3-one [91]. ORAC is a standardized method that integrates the degree and duration of the antioxidant reaction and uses biologically relevant radicals; however, it is time-consuming, requires expensive equipment and is susceptible to pH and instrument-induced variability [91].

The DPPH assay is a free-radical scavenging method for the evaluation of the antioxidant capacity of substances. The odd electron of the nitrogen atom in DPPH is reduced upon receiving a hydrogen atom from antioxidants. The delocalization of the spare electron over the molecule does not allow the dimerization of DPPH, while simultaneously, it provides the substance with a violet color and maximum absorption at 517 nm in ethanol solutions. Antioxidants reduce DPPH and cause a loss of the violet color in a dose-dependent manner. DPPH is a rapid, simple and inexpensive assay and can be utilized in aqueous and non-polar organic solvents for both hydrophilic and lipophilic antioxidants; however, it is soluble only in organic solvents and is sensitive to some Lewis bases and solvent types as well as oxygen, and the time response curve for reaching the steady state is not linear, with different ratios of antioxidant/DPPH [93].

FRAP exploits the formation of the blue-colored ferrous–tripyridyltriazine complex, caused by the reduction of ferric ions (Fe^3+^) to ferrous (Fe^2+^) at low pH. The presence of antioxidants reduces the formation of the complex and the intensity of the blue color. FRAP values are obtained by comparing the changes in absorbance at 593 nm of unknown samples with the absorbance of standard mixtures containing known concentrations of ferrous ions, using a spectrophotometer [94]. The assay provides fast and reproducible results, but requires an aqueous testing system [95].

Folin–Ciocalteu is a colorimetric assay for the measurement of total phenolic antioxidants in gallic acid equivalents. Phenolic compounds transfer electrons to phosphomolybdic/phosphotungstic acid complexes in an alkaline medium. This results in the formation of blue complexes, which are determined spectroscopically at 760 nm. The chemistry of the assay is non-specific, and other oxidation substrates can have inhibitory (oxidants competing with the reagent and air oxidation) or additive effects (aromatic amines, high sugar levels and ascorbic acid). However, extraction protocols can eliminate up to 85% of ascorbic acid and other interfering substances. The assay is simple, reproducible and extensively used [96].

## 6. Plant-Derived Antioxidants

### 6.1. Spice- and Herb-Derived Antioxidants

According to the U.S. Food and Drug Administration (FDA), the term “spice” is used to define “any aromatic vegetable substance in the whole, broken, or ground form, except for those substances which have been traditionally regarded as foods, such as onions, garlic and celery; whose significant function in food is seasoning rather than nutritional; that is true to name; and from which no portion of any volatile oil or other flavoring principle has been removed” (21CFR101.22), whereas herbs are considered “dietary ingredients for use by man to supplement the diet by increasing the total dietary intake”. The antioxidants most commonly found in spices and herbs are phenolic acids (caffeic, p-coumaric, ferulic and neochlorogenic acid) and flavonoids (quercetin, luteolin, apigenin, kaempferol and isorhamnetin). Additionally, herbs contain phenolic diterpenes (rosmanol and carnosol), phenylpropanoids (thymol, carvacrol and eugenol) and volatiles (1,8 cineole and α,β pinene) [97].

Spices and herbs present the highest antioxidant contents among all the animal- and plant-based food categories [98]. Their intense antioxidant capacity is mainly due to their rich contents of phenolic compounds, namely, terpenes (e.g., thymol, carvacrol, rosmanol and carnosol), phenolic acids (e.g., ferulic acid, caffeic acid, p-coumaric and eugenol) and flavonoids (e.g., quercetin and catechin) [97,99], supported by a variety of alcohols, esters, aldehydes, ketones and ethers [100,101,102]. Τhe antioxidant compounds of spices and herbs act mainly as free radical scavengers, blocking the oxidation process in the stage of initiation [91,95]. In other cases, they exhibit supplementary metal-chelating or superoxide-chelating antioxidant activity (e.g., rosemary and cumin) [95]. Spices and herbs deriving from different parts of the plants, such as the leaves, the bark, the arils, the bulbs, the roots and the flowers, are food additives traditionally used in food systems for their flavor, taste and color [103]. In the last decades, plants especially from the Apiaceae and Lamiaceae families have been used in medicine, cosmetics and the food industry as extracts (soluble fractions derived from solvent extraction) or essential oils (volatile oils derived from steam distillation) [102,104]. Herbs and spices utilized for their antioxidant properties in meat systems, and their effects on lipid and protein oxidation and the organoleptic traits of meat, have recently been reviewed by Aminzare et al. [105] and Kalogianni et al. [106]. The most commonly used herbs and spices in meat and meat products are oregano, rosemary, sage, thyme, tea and black pepper.

#### 6.1.1. Oregano

Oregano essential oil is derived from the plant species *Origanum majorana* and *Origanum vulgare*, and its major antioxidant compounds are carvacrol, thymol, p-cymene, rosmarinic acid, α-thujene, α,β-pinene, *p*-coumaric acid and γ-terpinene [97,102,107]. It has been widely studied and used as a natural antioxidant directly added in meat and meat-based products (chicken and pork meat, chicken liver meat, lamb burgers, and sausages) or their packaging (foal steaks) [108,109,110,111,112,113]. Moreover, the addition of oregano essential oil in animal feed additives (rabbits, pigs and sheep) has improved the oxidative stability of the meat produced [114,115,116,117].

#### 6.1.2. Rosemary

Rosemary extract exhibits high antioxidant activity, as it contains plenty of non-volatile phenolic substances (carnosic and rosmarinic acid, carnosol, rosmanol, rosmariquinone, rosmaridiphenol, rosmadial, 12-methoxycarnosic acid, epi- and iso-rosmanol, and caffeic acid) [97,107]. Its high antioxidant capacity is associated with the presence of two –OH groups in carnosic and rosmarinic acids, which donate cations and chelate metals [97]. However, the antioxidant activity of rosemary essential oil is remarkably reduced, due to the low contents of the remaining phenolic substances (1,8-cineole, α,β-pinene, limonene and camphor) resulting from the steam-distillation process used for its production [100,102]. Rosemary’s antioxidant capacity has been proven in pork patties [118,119,120], ham [121], pork sausages [122], fresh beef meat [123] and chicken frankfurters [124].

#### 6.1.3. Sage

Sage extracts from *Salvia officinalis* and *Salvia fruticosa* contain a wide variety of antioxidant compounds such as carnosol, carnosic acid, rosmanol, apigenin, luteolin methyl carnosate, rosmadial, 9-ethylrosmanol ether, epirosmanol, isorosmanol and galdosol [99,125,126]. The sage essential oil mainly contains α-thujone, camphor, viridiflorol, 1,8-cineole and α-pinene and exerts antibacterial, antifungal and free-radical scavenging activity [100]. Sage extracts have been tested in meat products (e.g., liver pates, meatballs and sausages) and have presented protective effects against lipid and protein oxidation [127,128,129,130].

#### 6.1.4. Thyme

Thyme essential oils and extracts are derived from the leaves of the plant species *Thymus vulgaris*, *T. mastichina*, *T. caespititius* and *T. camphorate*. Thyme has been used for centuries as an aromatic and medicinal plant [131]. Its basic antioxidant phenolic components are thymol, p-cymene, carvacrol, γ-terpinene and linalool [132]. Although thyme essential oil is considered a powerful natural antioxidant for meat products (e.g., minced beef, chicken meat and chicken sausages) [133,134,135], its addition is not always well-accepted by consumers due to its intense aroma and flavor [97].

#### 6.1.5. Tea

Green and black tea extracts exhibit remarkable antioxidant activity [136]. Their leaves are rich in flavonoids and phenolic acids such as catechins, tannins, theaflavins and thearubigenes [107,137]. The main flavonoids of green tea belong to the group of catechins and include catechin, epicatechin, gallocatechin, epicatechin gallate, epigallocatechin and epigallocatechin gallate [137], as well as alkyl volatile compounds [138]. Black tea mainly contains theaflavins, thearubigenes and chlorogenic, caffeic, *p*-coumaric and quinic acids [97].

Tea catechins exhibit remarkable antioxidant capacity associated with their free OH^−^ groups and their action as radical scavengers, metal chelators and donors of cations [97,107,137]. The antioxidant effect of tea extracts on meat oxidative stability has been studied in situ via direct application or incorporation in packaging films, with various results for the organoleptic traits of meat products such as pork meat, patties and sausages, liver pate, bacon and frankfurters [111,118,122,139,140,141,142,143,144].

#### 6.1.6. Black Pepper

Black pepper (*Piper nigrum*) is a spice widely used for its characteristic flavor and aroma. Its major component, piperine, belongs to alkaloids and is responsible for its pungent taste [49,145]. It also acts as a natural antioxidant, binding free ROS, radicals and hydroxyls [145]. Apart from piperine, black pepper consists of phenolic acids (caffeic acid and *p*-coumaric acid), other volatiles (α,β-caryophylline, limonene α,β-pinene, camphene and cymene), phenylpropanoids (cinnamaldehyde and eugenol) and sabinene [49,97], which reinforce its antioxidant action. Black pepper’s evidenced antioxidant properties in pork and beef meat and meat products [101,146,147] and its executive position in global gastronomy are likely to contribute to its wider acceptability by consumers and the meat industry as a natural antioxidant.

#### 6.1.7. Other Herbs and Spices

Other herbs and spices rich in phenolic compounds with strong antioxidant capacity that have been applied in meat and meat products and could be suitable for industrial use are cloves, marjoram, savory, basil, anise, caraway seeds, licorice, cinnamon, coriander, ginger, turmeric, cumin, paprika and nutmeg [10,101,105].

### 6.2. Fruit-Derived Antioxidants

Fruits are considered excellent sources of antioxidants, vitamins, minerals and fibers, continuously increasing public interest in incorporating them as health-promoting components in human diets [148]. The substantial phenolic content and other antioxidants in fruits make them attractive alternatives to synthetic antioxidants for food preservation [148]. Whole, chopped or mashed fruits have been used raw (e.g., fruit purees) or processed (e.g., dried, powdered, extracts etc.) for this reason. Moreover, fruit by-products (skins, peels, seeds and pulp) have also been used to improve the antioxidant capacity of meat and meat products [148]. Each one of the exploited fruits exhibits distinct antioxidant profiles and properties. Fruits and products thereof that are most commonly utilized by the meat industry for their antioxidant properties are grapes, plums, berries (bearberries, cranberries and strawberries) and pomegranates. Applications of fruit-derived antioxidants and their effects on the lipid and protein oxidation of meat, and on meat products’ organoleptic traits are presented in Table 2.

#### 6.2.1. Grapes

Grapes and their products are rich in phenolic compounds including anthocyanins, resveratrol, quercetin, kaempferol, catechins, phenolic acids and procyanidins, with their content of phenolic compounds varying from 63 to 182 mg/100 g of fresh fruits [184,185,186]. Most grape phenolic compounds are contained in grape skins (resveratrol, anthocyanins and catechins) and seeds (procyanidins) [187]. The commercial grape skins and seed extracts applied in meat products usually originate from grape pomace, a by-product of the wine industry [185]. Grape antioxidants mainly act as free-radical scavengers, with complex compounds able to chelate transition metal ions [185]. Grape seed extract in cooked minced turkey breast [178], raw pork patties [154], and raw and cooked minced chicken meat [168] has proven antioxidant properties. Reham et al. compared the antioxidant activity of grape seed extracts (50, 200 and 1000 mg/kg) with 0.01% BHT in minced beef. After a ten-day storage period at 4 °C, the grape seed treatments showed a dose-dependent reduction of TBARS values as follows: control (no treatment) > BHT > grape seed extract at 50 mg/kg > grape seed extract at 200 mg/kg > grape seed extract at 1000 mg/kg [188].

#### 6.2.2. Plums

Plums are rich in vitamins, carotenoids, flavonoids and phenolic acids [189]. After studying 20 plum genotypes, Rupasinghe [189] found that their antioxidant capacity ranged from 105 to 424 mg ascorbic acid equivalents, and the total phenolic content ranged from 86 to 413 mg of gallic acid equivalents per 100 g of fresh fruits, with a high coefficient of correlation between them (r^2^ = 0.96). The pigmentation of plums is attributed to anthocyanins (cyanidin 3-rutinoside, cyanidin 3-glucoside and peonidin 3-rutinoside), whereas their color intensity is associated with the total phenolic content [190]. Other phenolic compounds found in plums are hydroxycinnamic acid (chlorogenic acid and neochlorogenic acid) and quercetin derivatives, whereas plum skins are rich in anthocyanins and neochlorogenic acid [190]. In the meat industry, dried plums and dried plum puree have been used for their antioxidant properties [155,158,159]. Concentrated plum juice and spray-dried plum powder in beef roasts, [155], plum extract puree in irradiated turkey breast rolls [191], and plum puree in low-fat beef patties [162] have been successfully used to avert lipid oxidation. Basanta et al. incorporated fiber microparticles (MPCs) derived from the peel and pulp of Japanese plums in breast chicken patties. The MPCs reduced the TBARS values by 50% and increased the FRAP and redness values over a 10-day storage period at 4 °C [176].

#### 6.2.3. Bearberries

Bearberry leaf extracts contain approximately 312 mg/g of polyphenolic substances [192] and 90.42 mmol of Trolox equivalents/g of dry weight (antioxidant activity measured with the 2,2′-azino-bis (3-ethylbenzothiazoline-6-sulfonic acid) (ABTS) radical cation assay) [193]. The leaves contain high concentrations of the glycosides arbutin (5–15%), methylarbutin (4%) and free aglycones, whereas ursolic acid, tannic acid, gallic acid, p-coumaric acid, syringic acid, galloylarbutin, gallo-tannins and flavonoids are detected in lower concentrations [194]. The substantial phenolic content of bearberry extract makes it a popular natural antioxidant in food systems [193]. Crude bearberry leaf extract in meat systems [194] and bearberry extracts in raw pork patties [154] reduced TBARS values without affecting the sensory properties.

#### 6.2.4. Cranberries

Cranberries are rich in phenolic compounds such as phenolic acids, flavonoids, anthocyanins, p-hydroxybenzoic acid and their derivatives [195]. Mature cranberries have a total phenolic content of 4745 mg/kg in gallic acid equivalents and total monomeric anthocyanin content of 111.0 mg/kg [196]. The antioxidant capacity of cranberries is associated with their phenolic and anthocyanin–anthocyanidin contents. The mean total antioxidant capacity of cranberries has been estimated at 12.61 and 17.48 mmol of Trolox equivalents per kg when measured by the ferric reducing ability of plasma (FRAP) and Trolox equivalent antioxidant capacity (TEAC) assays, respectively [196]. Cranberry powder in mechanically separated turkey (MST) and ground cooked pork reduced TBARS values by 81% over a storage period of 7 days at 2 °C in the cooked pork and by 84% over a period of 6 days at 2 °C in the MST, indicating that the flavanol aglycones of cranberries are effective antioxidants for use in meat systems [150,197]. 

#### 6.2.5. Strawberries

Strawberries are good sources of antioxidants, containing vitamins, anthocyanins, flavonoids, and phenolic acids. Pelargonidin 3-glucoside, pelargonidin 3-rutinoside, pelargonidin 3-glucoside-succinate, cyanidin 3-glucoside and cyanidin 3-glucoside-succinate, which are responsible for the red color of ripe fruits, are the most common anthocyanidin glycosides in strawberries [198,199]. According to Huang et al. [200], strawberries have TEAC values of 4.44 ± 0.45 mmol of Trolox/100 g of dry weight (DW), a total phenolic content of 2.72 ± 0.18 mg of gallic acid/g of DW, a total flavonoid content of 7.04 ± 0.59 mg of rutin/g of DW and a total anthocyanin content of 1.16 ± 0.12 mg of catechin/g of DW [200]. The consumption of strawberries has been associated with various health benefits due to their antioxidant content, mainly in the form of vitamin C, anthocyanins, flavonoids and ellagitannins [201,202]. Strawberries’ antioxidant compounds have been shown to exhibit preventive activity against inflammation, oxidative stress, cardiovascular disease, certain types of cancers such as esophageal cancer and hemangioma, metabolic disorders (e.g., type 2 diabetes) and neurologic syndromes such as Purkinje disease [201,202,203]. Cooked chicken patties treated with 5% and 10% strawberry extracts had reduced TBARS values compared to 2% BHT-treated patties [170].

#### 6.2.6. Pomegranates

Pomegranates’ peels, arils, seeds and juice can be exploited for their antioxidant capacity. Pomegranate juice has a high content of anthocyanins, with 3-glucosides and 3,5-diglucosides of delphinidin, cyanidin and pelargonidin being the dominant ones [204]. Pomegranates contain punicalagin (tannin), which reaches a concentration level of 1500–1900 mg/L in commercial juices [205]. Punicalagin acts as a free-radical scavenger and has been found to inhibit lipid oxidation in vitro [206]. Pomegranate pulp and peel extracts obtained with organic solvents were found to contain ca. 24 ± 2.7 and 249 ± 17.2 mg/g of phenolic compounds, 17 ± 3.3 and 59 ± 4.8 mg/g of flavonoids and 5 ± 0.7 and 11 ± 0.5 mg/g of proanthocyanidins, respectively [206]. The beneficial properties of pomegranates are attributed to their rich contents of anthocyanins, ellagic acid, ellagitannins, punic acid, flavanols, flavan-3-ols and flavones [207,208]. Pomegranates have been found to have antioxidant, antidiabetic, antibacterial, anti-inflammatory, antiviral and cytotoxic properties (e.g., against cancer cells in breast cancer) [207,208,209]. Additionally, they protect the cardiovascular system and enhance oral health. Pomegranate phenolics in cooked chicken patties [177], the dipping of chicken breast meat in 0.02% (*v/v*) pomegranate fruit juice phenolics [178] and pomegranate juice, and rind and seed powder extracts in ground pork meat [179] were effective against lipid oxidation. Turgut et al. added 0.5% or 1% pomegranate peel extract and 0.01% BHT in beef meatballs to assess their antioxidant activity during refrigerated storage at −18°C for 6 months. In both pomegranate peel extract treatments, the TBARS values were decreased compared to those for control samples, whereas the 1% treatment was the most effective one (1% < BHT < 0.5% < control). Additionally, the treatments reduced protein oxidation by inhibiting the accumulation of carbonyls and the loss of total protein solubility and sulfhydryl groups, without affecting the color and sensory properties [180].

#### 6.2.7. Other Fruits

Other fruits or by-products thereof that could be exploited in meat systems for their antioxidant properties include apples, pears, citrus fruits, bananas, tomatoes, carob, blueberries, blackberries and blackcurrants [148].

## 7. Legislative Framework for Antioxidants in Meat and Meat Products

The Codex Alimentarius Commission is the international standard-setting organization responsible for the regulation and establishment of the rules governing the use of natural antioxidant compounds, in relation to their functions and food categories. However, food regulatory systems and legislative frameworks regarding the utilization of natural antioxidants as food additives may vary between different countries [104]. In general, the antioxidants approved for use in the European Union (EU) as food additives are ascorbates, tocopherols, gallates, erythorbate, butyrates, lactates, citrates, tartrates, phosphates, malates, adipates, EDTA and rosemary extracts [210]. Among natural antioxidants, tocopherols and carotenoids are regarded as safe food additives that normally possess a ‘‘Generally Recognized as Safe (GRAS)’’ status with the FDA in the USA, or a quantum satis state (unrestricted dose) in EU legislation. However, due to the fact that the excessive addition of antioxidants (synthetic or natural) may lead to pro-oxidant effects, their use is legally controlled via allowable levels [104,211].

In the EU, Regulation EC 1333/2008 on food additives established a list of authorized food additives that was published in full in Regulation EU 1129/2011 [212,213]. In the latter regulation, antioxidants are listed by their names and E-numbers as “other food additives”, and information regarding the corresponding permitted maximum levels and restrictions/exceptions per food category are detailed. According to this regulation, extracts of rosemary (E 392), carotenes (E 160a), tocopherol-rich extracts (E 306), α-tocopherol (E 307), γ-tocopherol (E 308), δ-tocopherol (E 309), and annatto, bixin and norbixin (E 160b) [105,210] are currently the natural antioxidants authorized as food additives by the EU. Considering meat, specific natural antioxidants are only allowed in processed meat products (heat-treated and non-heat-treated meat) [213] as summarized in Table 3.

In the USA, natural antioxidants are included in the Food Additive Status List issued by the U.S. Department of Agriculture (USDA) [214]. For meat products, the maximum allowable level of tocopherols is 300 mg/kg (used in poultry products), adjusted according to the total fat content of the meat, and they should not be used in combination with other antioxidants. Despite the Codex Alimentarius Commission’s opinion for a maximum carotenoid level of 20 mg/kg in fresh meat, poultry and game (comminuted) [215], this proposal is not adopted by the USA legislation.

## 8. Application of Natural Antioxidants in Meat Industry

The conventional method of incorporating natural antioxidants into meat products has been proven to be more effective than spraying them on their surface [216]. The direct contact of antioxidant substances with the meat is desirable for their efficient action; however, the exposure to environmental conditions, such as intense light, oxygen, high pH and temperature, may precipitate the inactivation of their antioxidant capacity [216]. Novel approaches, such as active packaging and edible coatings/films, exploit the beneficial effects of antioxidants, avoiding the concomitants of mixing with the meat product, their destabilization and the commercially undesirable effects on meat quality traits (color, flavor and taste). Moreover, these technologies improve the cost–benefit ratio of the addition of natural antioxidants in meat systems, due to the reduction in the quantities of antioxidants required and the capability of avoiding the questionable practice of incorporating antioxidants into the meat product [217,218].

According to the Commission Regulation No 450/2009 [219], active packaging refers to “materials and articles that are intended to extend the shelf-life or to maintain or improve the condition of packaged food; they are designed to deliberately incorporate components that would release or absorb substances into or from the packaged food or the environment surrounding the food”. Natural antioxidants (tocopherols, plant extracts and essential oils) are mainly used as intrinsic components of the packaging container and polymer film, expressing their antioxidant properties by gradual release in the packaged meat product [220].

Although edible coatings and films are not included in the definition of active packaging, they act similarly, closely surrounding the meat product and releasing the active antioxidant agent directly on its surface [218,221]. Edible films and coatings are constructed by mixing biopolymers such as lipids, proteins and polysaccharides, and the natural antioxidant compounds in the form of extracts or essential oils [217,218]. The antioxidant capacity of the edible coatings or films is determined both by the interaction between their components and the chemical composition of the meat product [218].

Nanoparticles constitute an emerging technology and a promising tool for the efficient application of natural antioxidants in meat products, as they minimize the quantity of active substances required. Nano-encapsulation contributes to the most effective incorporation of natural antioxidants in packaging materials and films by (i) increasing the functionality of natural antioxidants during meat product life and storage, (ii) protecting antioxidants from extreme environmental conditions and thermal processing in the case of edible coatings/films, and (iii) interacting with the chemical and mechanical properties of the polymer matrix [222].

The oxidative stability of meat and meat products can be improved through the addition of natural antioxidants in animal feedstuffs. Many studies have demonstrated the beneficial results of adding antioxidants in feedstuffs for both the health and performance of animals, and the quality of the derived meat products. The incorporation of α-tocopherol in pig [223], cattle, sheep and poultry diets [107] delayed lipid oxidation and preserved the meat’s color during its storage. Adding rosemary [224,225], thyme [226] and oregano oil [115], pomegranate by-products [227], grape seeds [228] and pomace [229], and licorice extract [230] into sheep diets improved the antioxidant capacity and organoleptic traits of the derived meat. Similarly, a mix of oregano essential oil and sweet chestnut wood extract [116], olive leaves [231] and a plant extract of *Lippia* spp. [232] in pigs’ diets improved the meat oxidation status and consumer acceptance after cooking. In broilers, the addition of grape by-products that were rich in polyphenols improved the growth rate [233] and reduced meat lipid oxidation [233,234]; similar results were observed with sage, rosemary, oregano oil [107] and *Moringa oleifera* leaves [235]. Furthermore, feeding rabbits with chestnut tannins had a beneficial effect on growth rates, welfare and meat antioxidant capacity [236], whereas thyme leaves increased the oxidative stability and color acceptance of rabbit meat [237]. In general, plant-derived natural antioxidants, when used as feed supplements in animal diets, enriched with polyunsaturated fatty acids, can be useful for the prevention of lipid oxidation and the preservation of high-quality meat traits [238].

## 9. Future Challenges for and Limitations of Natural Antioxidant Use in Meat Systems

Time-, storage- and treatment (e.g., cooking, heating, seasoning etc.)-dependent changes in the antioxidant profiles set limitations on their universal and unquestionable use. The potential health benefits from integrating antioxidant additives in meat systems are not always proven. Therefore, the meat industry is mainly motivated by the positive effects of antioxidants on the shelf-life and the safety and quality assurance of meat and meat products and advertises the potential health benefits for the consumers on a secondary basis.

When evaluating a plant-derived compound to be utilized for its antioxidant capacity in meat systems, it is critical to consider the compound’s physicochemical traits (e.g., fat solubility, optimum concentration and temperature, pH and thermal stability), as well as other factors including its cost, availability and regulatory status [239]. Further in vitro investigation is critical in order to reveal potential unknown benefits and/or drawbacks associated with the utilization of natural antioxidant compounds in meat systems; at a certain stage, direct in situ studies on the concerned foods under natural conditions need to be undertaken to confirm or reject the initial claims. 

The improvement of current technologies and the development of new ones for the incorporation of natural antioxidants in meat systems is a prime objective for their mass industrial use. The harmonization of methods with consumer demands and the meat industry’s capabilities, and an evidence-based gradual exploitation of natural antioxidants are necessary to secure a genuine transition in the field.

Moreover, major challenges for the wider application of plant-derived antioxidants in meat systems are (i) the increased processing and storage costs of plants/fruits and the derived antioxidants, (ii) the marginal and constantly compressed profit from meat trading and (iii) the increased amounts required in order to exhibit efficient antioxidant action (compared to synthetic antioxidants), which could result in alterations of the meat’s organoleptic traits (e.g., taste, color and odors) and, therefore, a decrease in its marketability. 

The overall aim is to provide meat products with a tailored quantitative and qualitative antioxidant profile, to allow the precise addition of natural antioxidant compounds and, thereby, the most efficient exploitation of their beneficial effects on preservation, organoleptic traits, consumer health and acceptance. Clinical trials are necessary to evaluate the safety of natural antioxidant substances, and to confirm health claims and perceptions. However, clinical trials are time-consuming and expensive, and the profit margins of the meat industry do not justify their funding without the marketing and promotion of antioxidant-functionalized meat products as a prime target. In this framework, clinical trials studying the incorporation of natural antioxidants (purified substances or extracts) in meat systems and their effects on human health are scarce and often inconclusive.

## 10. Conclusions

Numerous in vitro studies have demonstrated the positive effects of natural antioxidants on lipid and protein oxidation, the prolongation of shelf-life, the antioxidant profiles of functionalized meat products, and the related potential health benefits. Additionally, the analysis of the antioxidant profiles and the course of action of plant-derived food additives, as well as the investigation of perspectives for applications in the meat industry, has made significant progress. Factors that limit the wider acceptance of natural, plant-derived antioxidants include (i) the fragmentation of the legislative framework regarding the use of food additives on a global scale, which obstructs international trade; (ii) the complex matrices used in applications of natural antioxidants (e.g., extracts, powders, purees etc.) and their interactions, which do not allow the definite characterization of the actual antioxidant substances and the quantification of their antioxidant capacity; (iii) the questionable usability and profitability of natural antioxidants and the scarcity of universally accepted, effective application technologies in meat systems that do not deteriorate the meat’s organoleptic traits; and (iv) the need for clinical studies and safety tests to evidence the potential health claims and eliminate the risk of undesirable effects. Novel approaches and state-of-the-art technologies such as active packaging, edible coatings and nanoparticle technology could provide sustainable alternatives for the functionalization of foods by adding plant-derived antioxidants. 

## Figures and Tables

**Table 1:** Classification of phenolic compounds based on their carbon chains.

Class	Basic Skeleton	Plant Sources
Phenols and benzoquinones	C6	*Primula obconica* [37], sorghum [38], berries, fruit wines, olive oil [39]
Phenolic acids	C6–C1	blueberries, blackberries, persimmon, apple juice, cider, cherry laurels, canola meal, oranges, rye [39]
Acetophenones and phenylacetic acids	C6–C2	root bark of *Derris indica*, *Ageratina pichinchensis* [40], spruce, pine [41], pisum, nicotiana, phaseolus, triticum, trapoleum, lycopersicon, avena [42], balsamic vinegar [43], cocoa powder [44]
Hydroxycinnamic acids, phenylpropenes, coumarins-isocoumarins and chromones	C6–C3	berries, pomes, herbs, seeds, cereal grains, leafy greens, asparagus, cinnamon, cloves and potatoes [45]
Naphthoquinones	C6–C4	oranges, Plumbaginaceae, Droseraceae, Ebenaceae [37]
Xanthones	C6–C1–C6	*Gentianna ottonis* [39], Guttiferae, Moraceae, Clusiaceae, and Polygalaceae [46]
Stilbenes and anthraquinones	C6–C2–C6	grapes, pine, peanuts, sorghum, rheum [47], Rubiaceae [48], black pepper [49]
Flavonoids (flavones, flavonols, flavanones, flavan-3-ols, isoflavones, anthocyanidin compounds)	C6–C3–C6	celery, parsley, red prickly pears, olives, acerola, litchis, avocadoes, green and black tea, cherries, raspberries, strawberries, grapes and red wine [45]
Lignans and neolignans	C6–C3	wheat, oats, rye, barley [50], berries [51]
Lignins	(C6–C3)n	*Arabidopsis thaliana*, *Pinus radiata* [52], sugar cane [53], spruce, wattle, birch, rice, eucalyptus, pine [54]

**Table 2:** Effects of fruit-derived antioxidant compounds in meat and meat-based products.

Meat/Meat-Based Product	Treatment (Main Antioxidant Compound)	LA ^1^	PA ^2^	COL ^3^	FL/TA ^4^	FA ^5^	TBARS ^6^	Ref ^7^
Pork meat and meat-based products								
Ground meat	Pomegranate rind powder extract	++	N/A	0	+	BHT (+)	++	[149]
	Pomegranate juice, seed powder extract	+	N/A	0	+	BHT (+)	+	
	Cranberry powder (phenolic acids, anthocyanins, flavanols and proanthocyanidins)	++	N/A	N/A	N/A	N/A	++	[150]
Patties	Red grape pomace extracts	++	N/A	+	N/A	N/A	++	[151]
	Grape seed extract (N/A)	++	N/A	++	N/A	ΒHΤ (−/0)	++	[152]
	Blackcurrant (*Ribes nigrum* L.) extract (anthocyanins)	++	++	0	N/A	BHA (0)	++	[153]
	Grape seed extract (N/A)	++	N/A	0	0	Ν/A	++	[154]
	Bearberry extract (N/A)	++	N/A	0	0	Ν/A	++	
Liver pâté	Grape extract (N/A)	++	N/A	0	N/A	ΒHΤ (−)	++	[141]
Sausages	Plum puree	++	N/A	0	−/0	BHA/BHT	++	[155]
	Plum and apple puree	++	N/A	0	−/0	BHA/BHT	++	
	Banana male flower extract (flavonoids)	++	N/A	0	0	N/A	++	[156]
	Grape seed extract (N/A)	++	Ν/A	+	+	BHT (−)	++	[157]
Ham	Plum juice concentrate, plum powder	0	N/A	0	−/0	N/A	0	[158]
Beef meat and meat-based products								
Roast beef	Plum juice concentrate, plum powder	++	N/A	0	0	N/A	++	[159]
Ground beef	Commercial grape seed extract	+	N/A	N/A	0	BHA/BHT (−)	+	[160]
Patties	Grape seed extract	+	N/A	0	0	N/A	+	[161]
	Plum puree	+	N/A	0	−/0	N/A	+	[162]
	Dried plum puree	++	N/A	N/A	N/A	BHA/BHT (+)	++	[163]
	Seasonings derived from wine pomace (N/A)	++	N/A	N/A	N/A	Sulfites (−)		[164]
Meatballs	Lyophilized pomegranate peel nanoparticles	++	N/A	+	+	BHT (−)	++	[165]
	Pomegranate peel extract (N/A)	++	++	+	0	BHT (−)		[166]
Sausages	Grape seed extract (N/A)	++	N/A	+	+	AA, PG (−)	++	[167]
Poultry meat and meat-based products								
Fresh meat	Grape seed and peel extracts	+	N/A	(−/0)	(−/0)	BHT (0)	+	[168]
	Dipping in pomegranate juice phenolic solution	+	+	N/A	+	N/A	+	[169]
Cooked chicken breast meat	Prunus mume (Japanese apricot) methanolic extracts	++	N/A	0	N/A	N/A	++	[170]
Chicken meat wafer	Apple peel (N/A)	++	N/A	+	+	N/A	++	[171]
	Banana peel (N/A)	++	N/A	+	+	N/A	++	
Chicken patties	Grape dietary fiber	++	N/A	0	+	N/A	++	[172]
	Grape seed extract + NaCl 1%	++	N/A	N/A	N/A	N/A	++	[173]
	Pomegranate juice, pomegranate rind powder extract (N/A)	++	N/A	0	0	BHT (−)	++	[174]
	Pomegranate peel powder and extract	++	Ν/A	−	N/A	BHT (−)	++	[175]
	Plum peel and pulp fiber microparticles (β-carotene, lutein, α- and γ-tocopherols, polyphenols)	++	Ν/A	0	0	Ν/A	++	[176]
Chicken meatballs	Pomegranate rind powder extract	+	N/A	(0/+)	(0/+)	BHA/BHT (−)	+	[177]
Turkey meat	Grape seed extract	++	N/A	N/A	N/A	N/A	++	[178]
	Cranberry powder (phenolic acids, anthocyanins, flavanols and proanthocyanidins)	++	N/A	N/A	N/A	N/A	++	[150]
	Peach skin powder	++	N/A	N/A	N/A	BHA (0)	N/A	[179]
Lamb meat and meat-based products								
Patties	Red grape by-product extract (N/A)	++	++	+	N/A	SA (−)	++	[180]
	Pomegranate by-product extract (N/A)	0	0	0	N/A	SA (0)	0	
Sheep meat nuggets	Litchi pericarp extract	+	N/A	N/A	0	BHT (0)	+	[181]
Goat meat and meat-based products								
Ground meat	Grape seed extract	+	N/A	+	N/A	TBHQ (+)	+	[182]
	Tea extract	+	N/A	−	N/A	TBHQ (+)	+	
	Pomegranate peel extract + vacuum packaging	+	N/A	0	0	N/A	+	[183]
Goat meat nuggets	Pomegranate peel extract + vacuum packaging	+	N/A	0	0	N/A	+	[183]

^1^ lipid antioxidant effect, ^2^ protein antioxidant effect, ^3^ color effect, ^4^ flavor/taste effect, ^5^ food additives other than phenolic compounds, ^6^ thiobarbituric acid reactive substances assay (TBARS), ^7^ reference. In FA column, 0, + and − indicate equal (0), increased (+) or decreased (−) antioxidant capacity of synthetic food additives compared to natural phenolic compounds. In columns LA, PA, COL, FL/TA and TBARS, the symbols indicate +: poor positive effect, ++: strong positive effect, −: negative effect, and 0: no effect (the grading system reflects the opinions/conclusions of the corresponding referenced citations). AA: ascorbic acid, BHT: butylated hydroxytoluene, BHA: butylated hydroxyanisole, TBHQ: tertiary butylhydroquinone, SN: sodium nitrite, TC: α-tocopherol, SC-SE: sodium citrate–sodium erythorbate, PG: propyl gallate, SA: sodium ascorbate, N/A: relevant data are not available.

**Table 3:** Maximum levels of natural antioxidants permitted by the EU in meat and meat products.

Food Category	E-Number	Name	Maximum Level (mg/kg)	Restrictions/Exceptions
Non-heat-treated and heat-treated processed meat	E 160a	Carotenes	20	Only sausages, pâtés and terrines
	E 392	Extracts of rosemary	100	Only dried sausages
			150	Excluding dried sausages
			150	Only dehydrated meat
